# Supplementary material for: Mapping the loss of flagellar motility across the tree of life
Source: ISME J. 2025 Jun 13;19(1):wraf111. doi: 10.1093/ismejo/wraf111 (PMC12199754; doi:10.1093/ismejo/wraf111)
Supplement: ISMEJ_Philip_SI_provAcc_20250527_wraf111 [file ismej_philip_si_provacc_20250527_wraf111.pdf]

Supplementary information for "Mapping the loss of flagellar motility across the tree of life"

Jamiema Sara Philip, Sehhaj Grewal, Jacob Scadden, Caroline Puente-Lelievre, Nicholas J. Matzke, Luke McNally, and Matthew AB Baker.

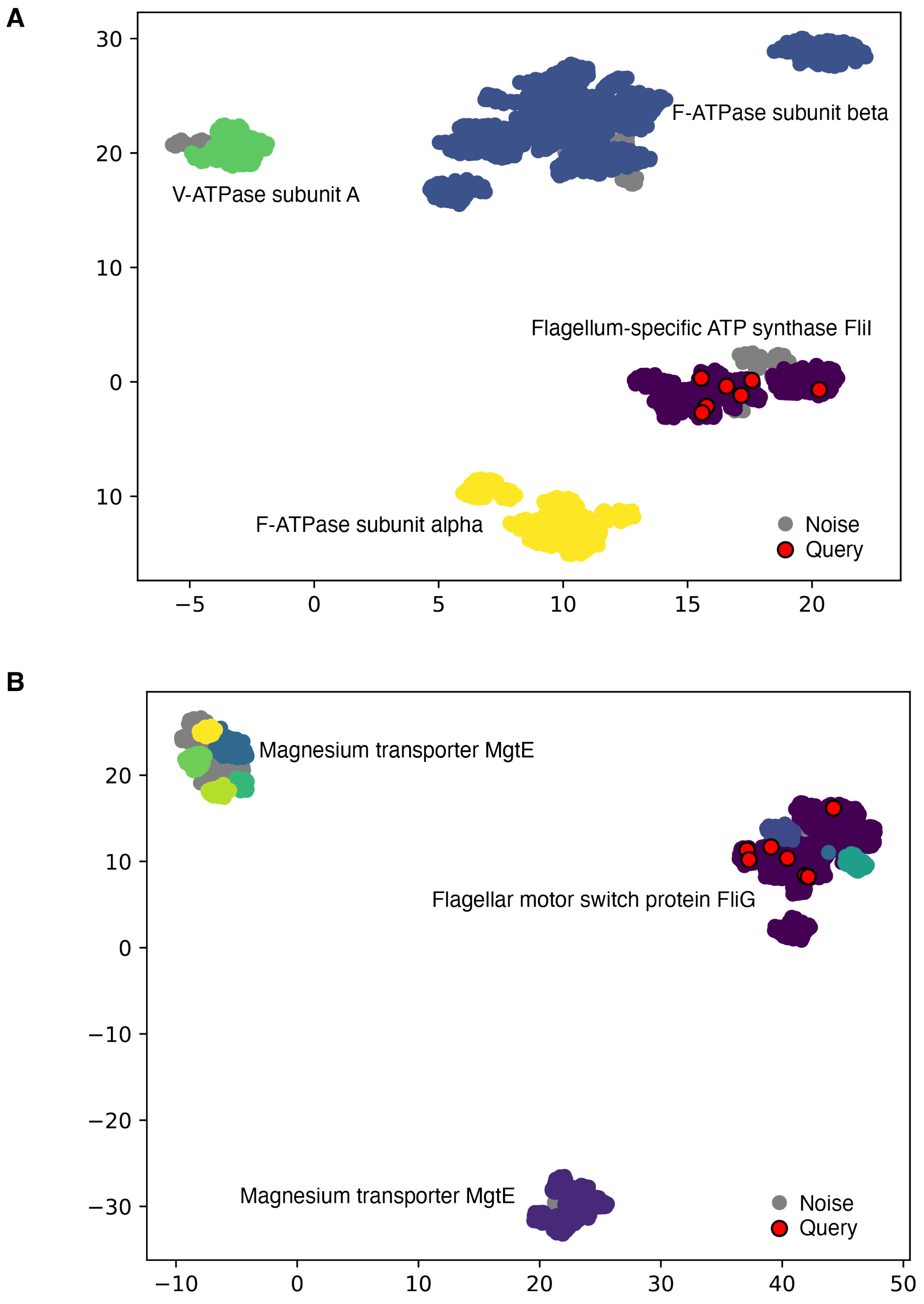

Supplementary Fig. S1. DBSCAN clustering of UMAP embeddings for (A) FliI and (B) FliG. Pairwise distance matrices from multiple sequence alignments were reduced to low-dimensional embeddings using UMAP and clustered with DBSCAN. Cluster annotations were assigned based on information retrieved from UniProt using ID mapping between PATRIC genome IDs and UniProt IDs. Each point represents a protein sequence, with colours indicating different clusters.

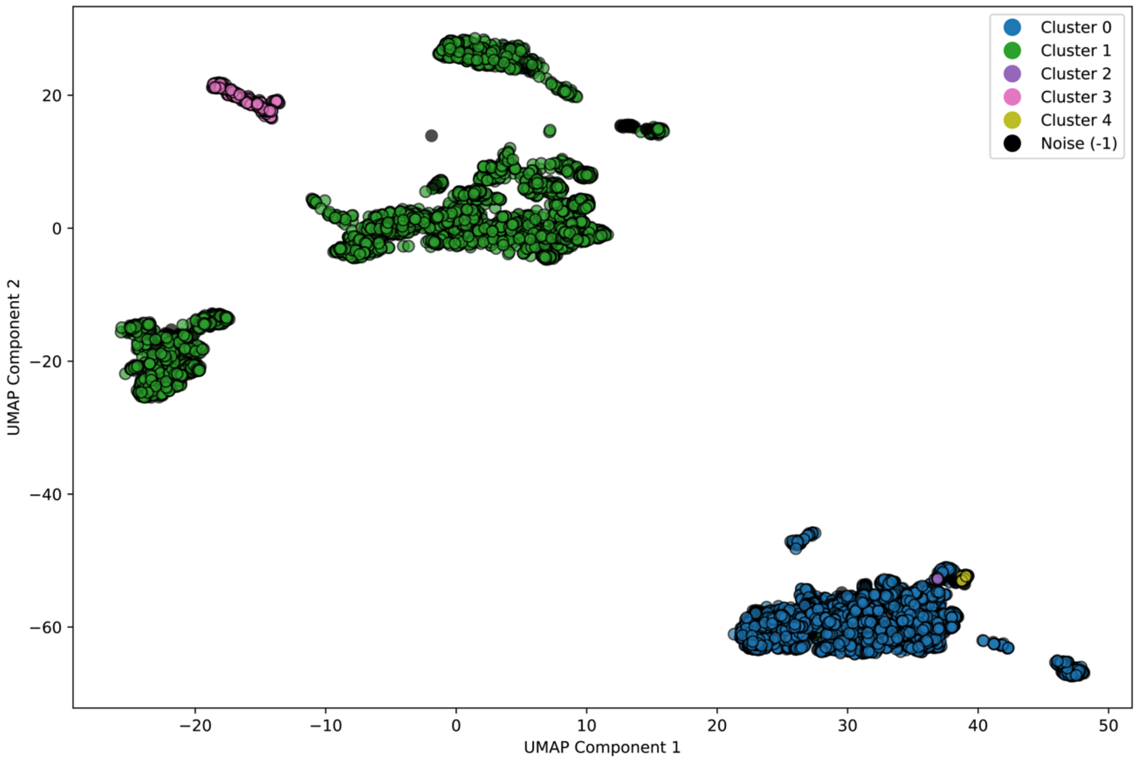

Supplementary Fig. S2. DBSCAN clustering of UMAP embeddings for the motility classifier. The distance matrices based on the presence-absence of flagellar proteins across genomes were reduced to low- dimensional embeddings using UMAP, and then clustered using the DBSCAN.

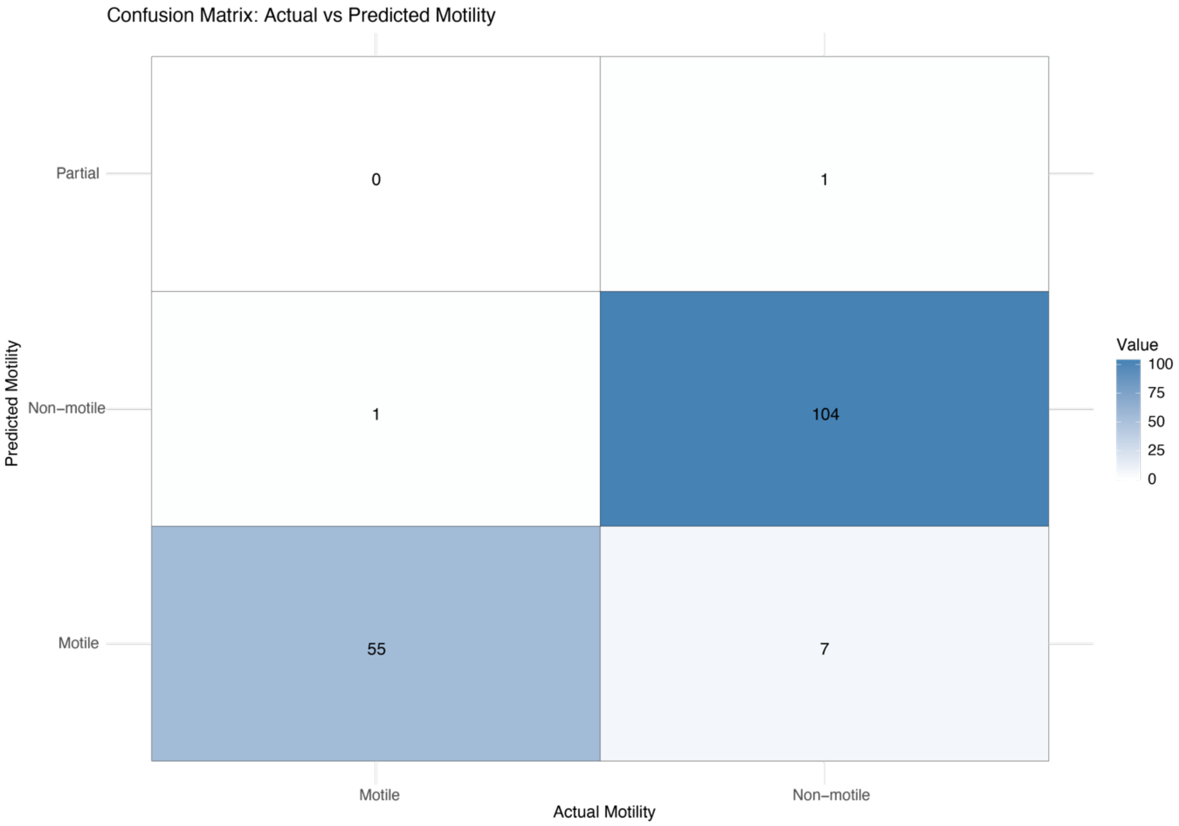

Supplementary Fig. S3. Confusion Matrix of Motility Classifications. This confusion matrix compares the motility classifications of 169 species from our classifier dataset with those from the Firmicutes motility dataset [13].

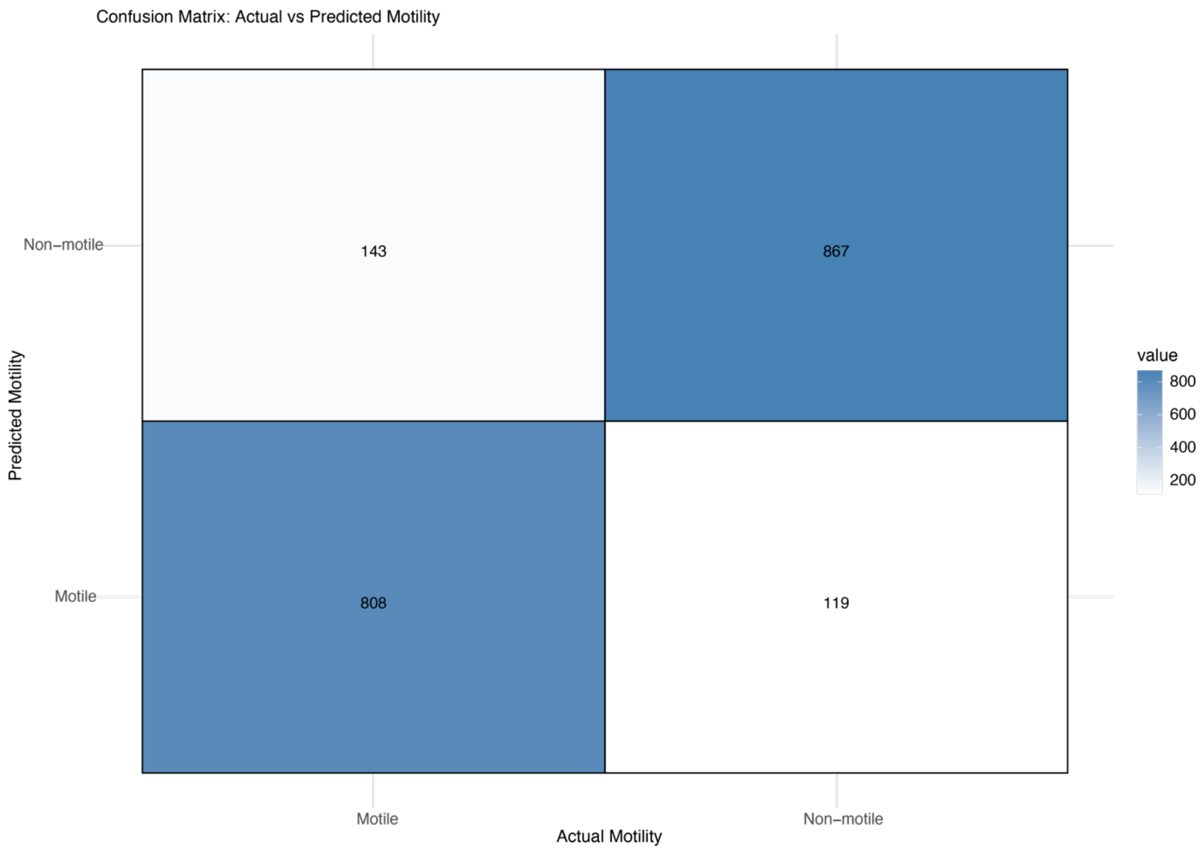

Supplementary Fig. S4. Confusion Matrix of Motility Classifications. This matrix compares the motility classifications of 1937 species in our classifier dataset with those from a previous study (21).

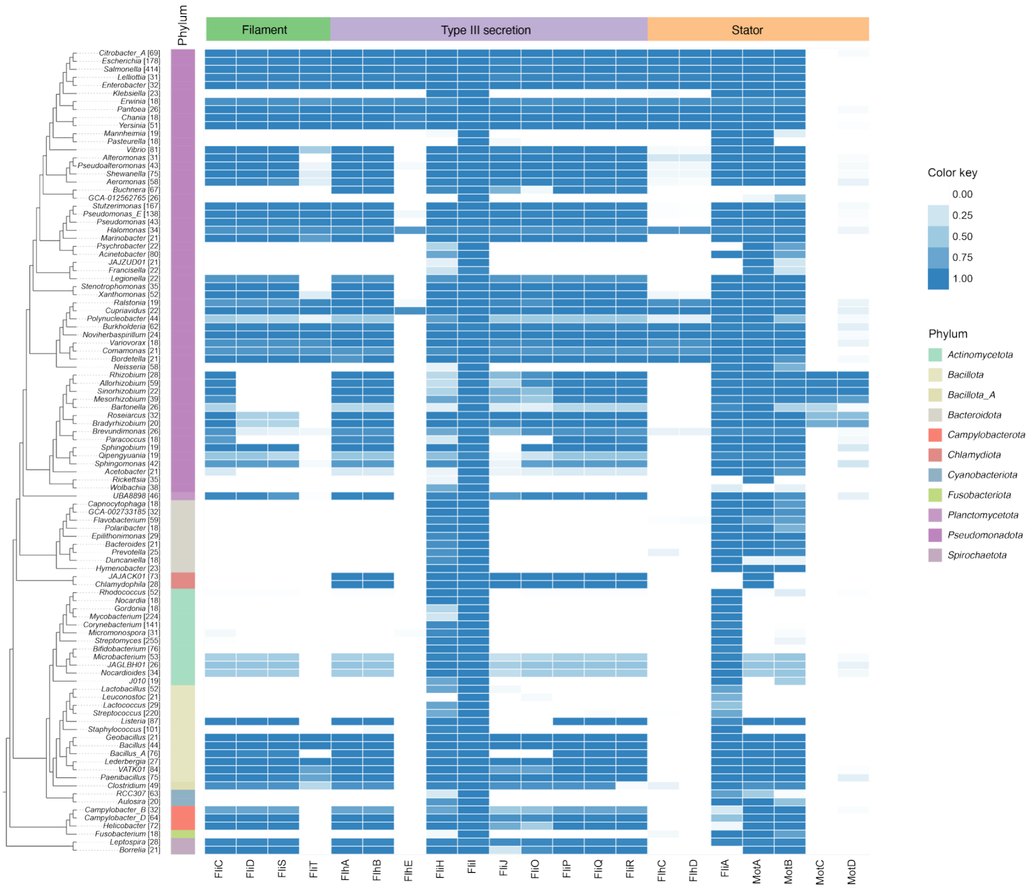

Supplementary Fig. S5. Heatmap shows the detailed breakdown of the normalized abundance of flagellar protein homologues, specifically filament, Type-III Secretion System (T3SS), and stator proteins, across the most prevalent genera (Fig. 2). Data were derived from species with available GTDB lineage information. Presence-absence counts were aggregated at the genus level and normalized based on both the number of strains within each genus and the number of proteins in each functional class (Fig. 1C). The adjacent cladogram illustrates the GTDB taxonomy of the top 100 most represented genera, selected from the 4,125 species included in the classifier (Fig. 3). The tree was pruned from the GTDB-reference tree by selecting a single representative species for each of the selected genera. Color intensity corresponds to the relative presence of flagellar proteins with higher values indicating greater abundance in that genus.

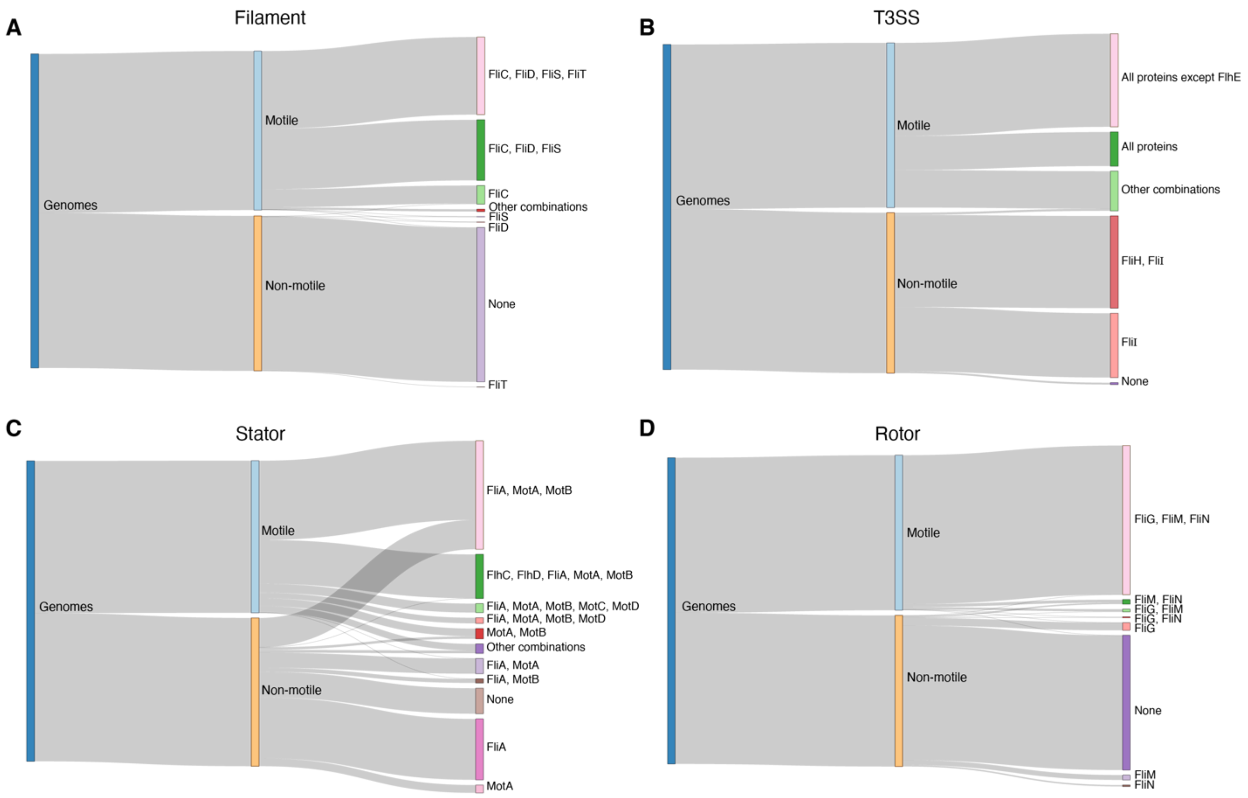

Supplementary Fig. S6. Sankey plot showing the distinct patterns of flagellar proteins across motile and non-motile genomes for each flagellar component: A) Filament, B) Stator, and C) Type III secretion system (T3SS), D) Rotor. In each panel, genomes are first divided into *motile* and *non-motile* categories. Subsequent branches display individual proteins/different combinations associated with each component of the flagellar apparatus. *Other combinations* indicate under-represented combinations not shown individually, whereas *None* represents genomes lacking those proteins. The width of each flow is proportional to the number of genomes.

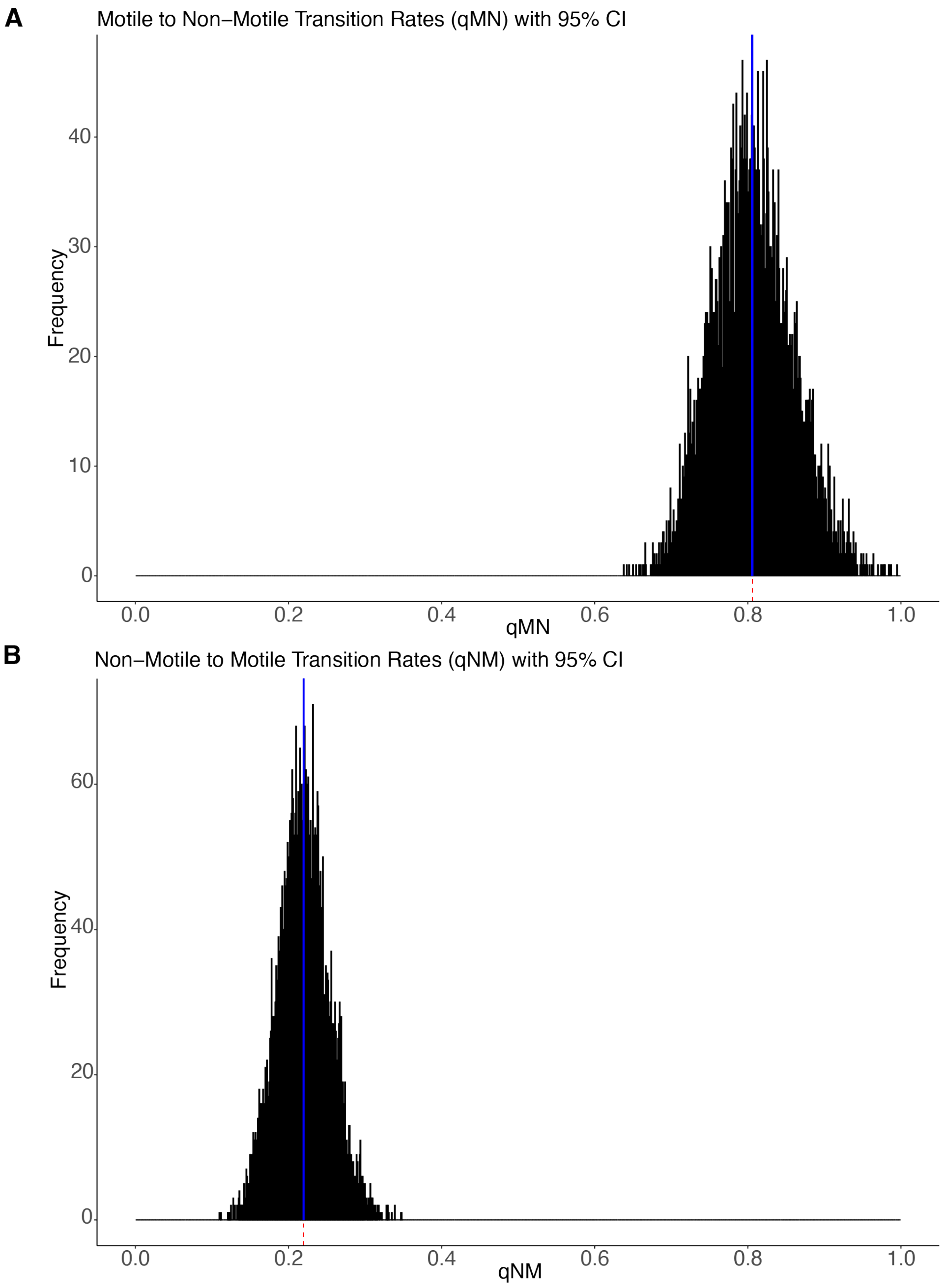

Supplementary Fig. S7. Transition rates of motility. Histograms indicate the posterior distribution of transition rate estimates between motile (qMN) and non-motile qNM). The red dotted line indicates the mean, and the blue line indicates the 95% CI.

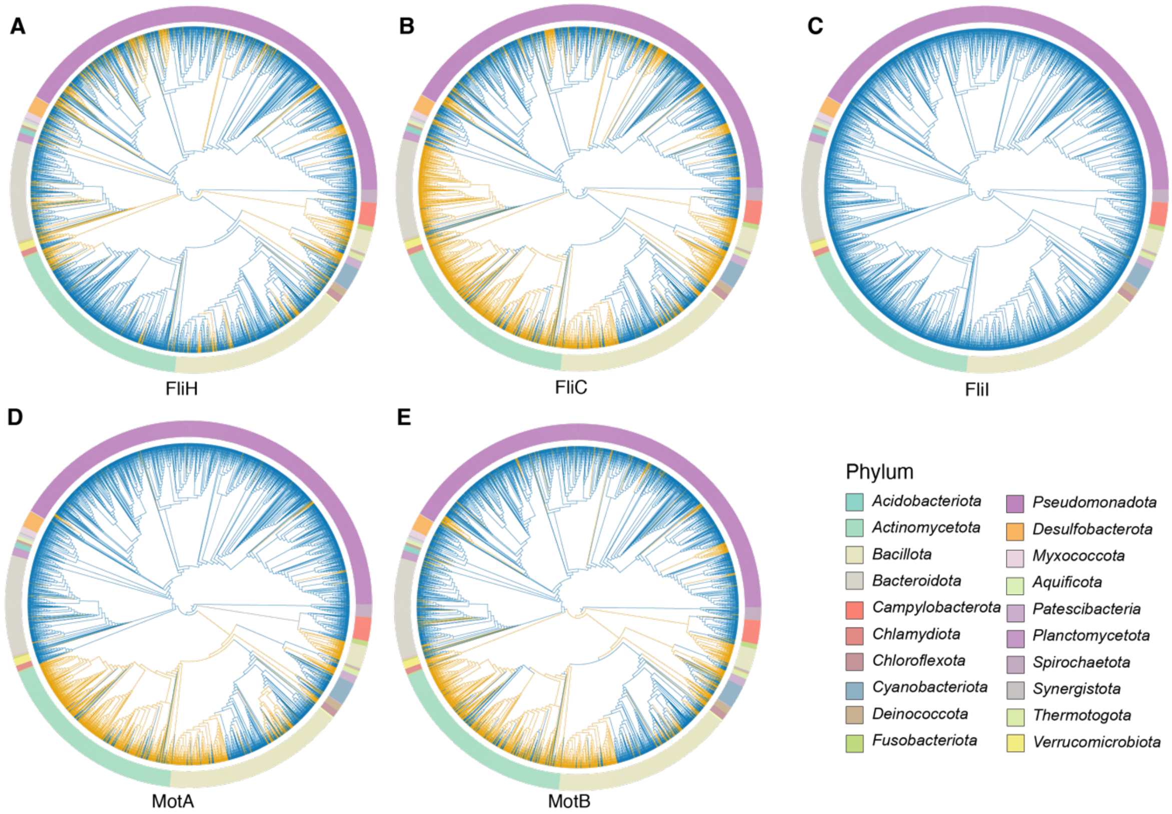

Supplementary Fig.S8: Bayesian ancestral state reconstruction of the gain and loss of flagellar proteins across the bacterial phylogeny: (A) FliH, (B) FliC, (C) FliI, (D) MotA, and (E) MotB. The phylogenetic tree depicts the relationships among 4,125 bacterial species common to our dataset and the Genome Taxonomy Database (GTDB). This tree is pruned from the GTDB reference tree for the matching 4125 bacterial species. Branches are colored according to the posterior probability of protein presence (gain) or absence (loss) at each node, as estimated using the Markov Chain Monte Carlo (MCMC) multistate model in Bayestraits. Blue indicates a higher probability of protein presence whereas orange indicates a higher probability of protein absence.

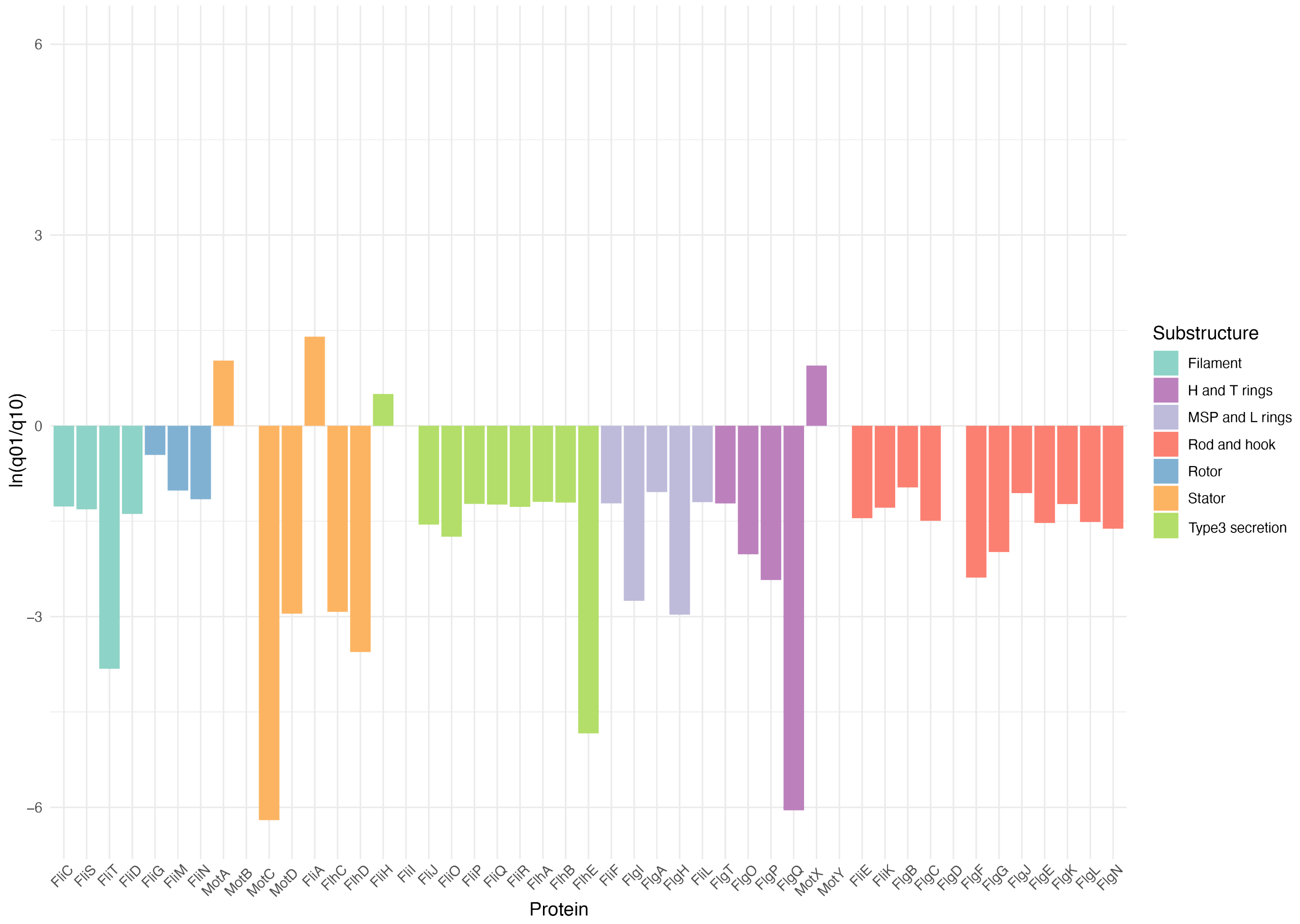

Supplementary Fig. S9: Bar Plot showing the log-transformed gain/loss ratio (ln(q01/q10)) for each flagellar protein, with colors indicating their associated flagellar substructures. Positive values indicate a higher frequency of gain events, whereas negative values indicate a higher frequency of loss events. Values close to zero indicate similar rates of gain and loss.

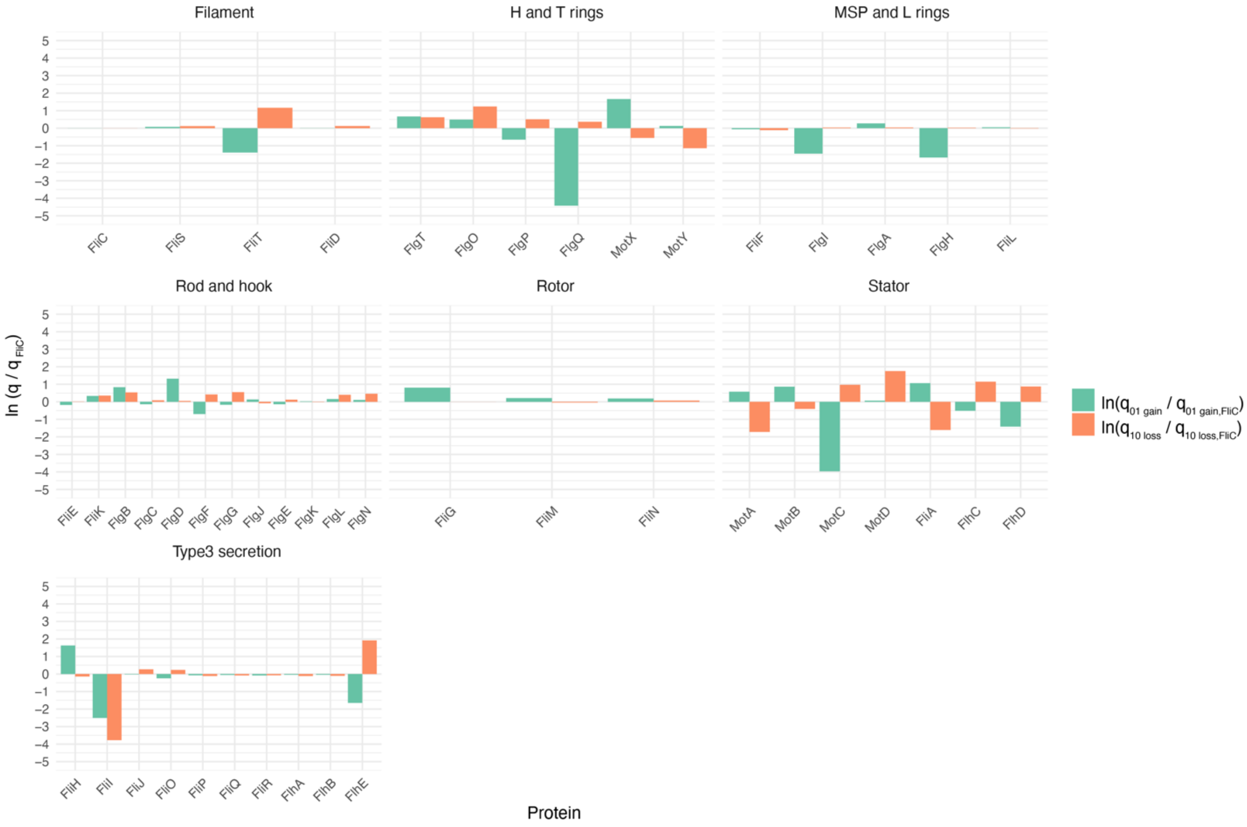

Supplementary Fig. S10: Bar Plot showing the log-transformed normalized gain and loss rates for each protein relative to FliC. The y-axis represents **ln (q_{01_gain} / $q_{01\_gain,\ FliC}$) or (q_{10_loss} / $q_{10\_loss,\ FliC}$))**, where q_{01_gain} and q_{10_loss} indicate the gain or loss rates of a given protein, normalized against the respective rates for FliC. A **Positive value** indicates a higher gain rate compared to FliC and **negative value** indicates a lower gain rate compared to FliC whereas **zero** indicates same gain rate as FliC. Bars are grouped by proteins within different substructures, with separate bars for gain and loss rates.

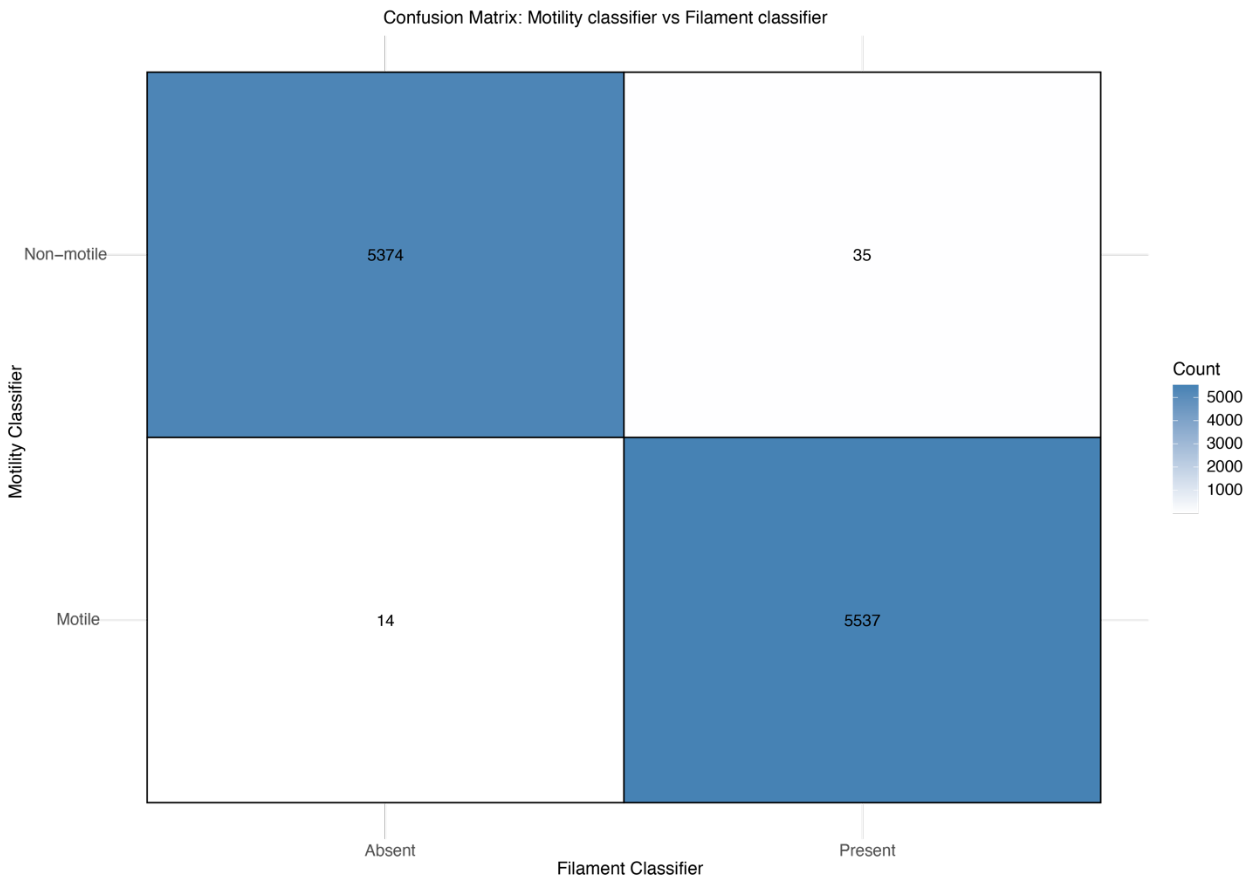

Supplementary Fig. S11. Confusion Matrix of Filament Classifications. This matrix quantifies presence and absence of filament proteins (FliC, FliD, FliS, FliT) for motile and non-motile clusters for 10960 species present in our dataset (full dataset with those genomes classified as partial removed). Accuracy is calculated at 99.6% as: Accuracy = (TP + TN) / (TP + TN + FP + FN)

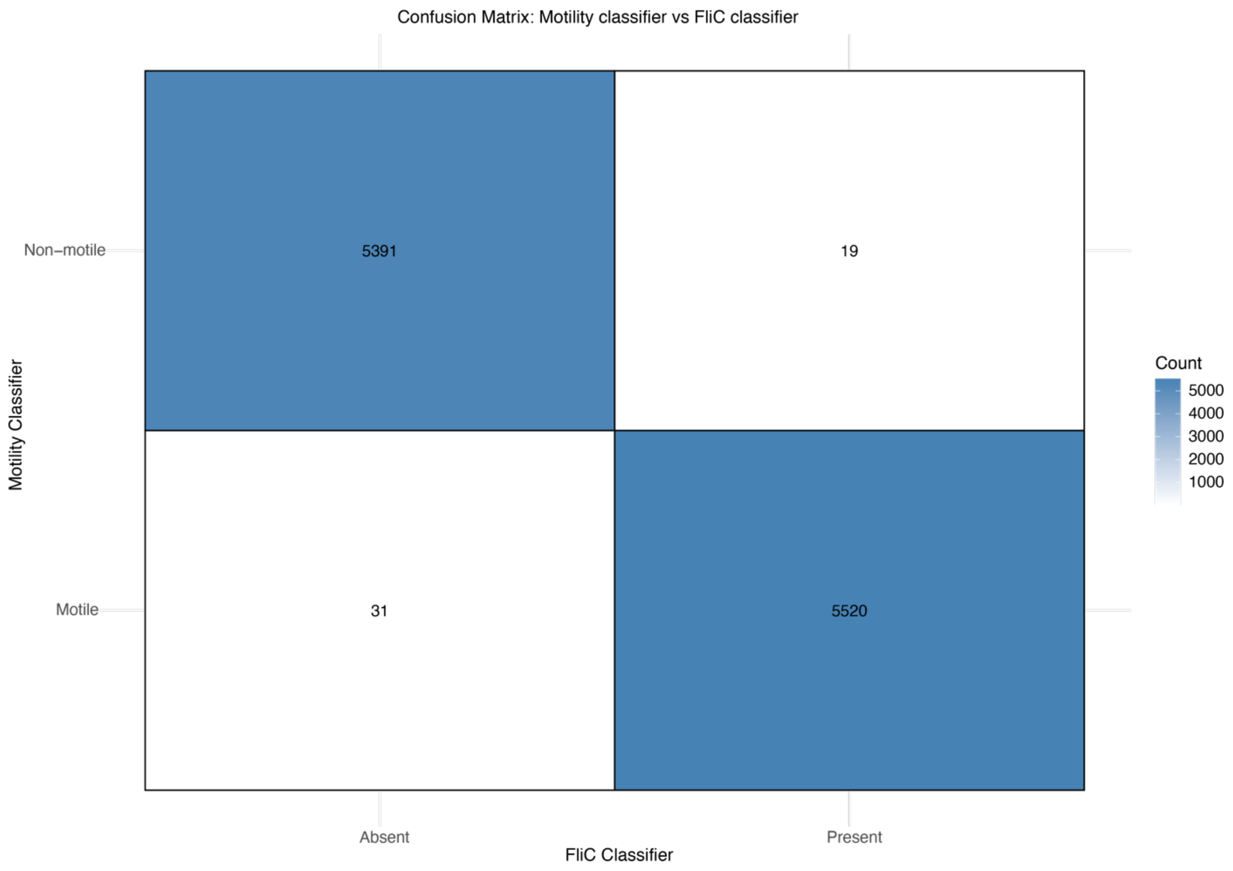

Supplementary Fig. S12. Confusion Matrix of FliC *Classification.* This matrix quantifies presence and absence of FliC or motile and non-motile clusters for 10960 species present in our dataset (full dataset with those genomes classified as partial removed). Accuracy is calculated at 99.5% as: Accuracy = (TP + TN) / (TP + TN + FP + FN)

Supplementary Table S1: List of species and their corresponding PATRIC genome IDs used in this study.

Supplementary Table S2: The complete list of species and flagellar proteins, together with the total number of proteins present.

Supplementary Table S3: Table showing the percentage of sequences removed for each flagellar protein during the length-filtering step.

	Multistate (*Log ML*) (qMN≠qNM)	Multistate (*Log ML*) (qMN=qNM)	Log BF
Motility	-1098.5479	-1136.74637	76.396876
**Log BF = 2(log marginal likelihood complex model (qMN≠qNM) – log marginal likelihood simple model (qMN=qNM)**			

Supplementary Table S4: Log Marginal Likelihood (log ML) estimates using the BayesTraits Multistate algorithms for equal and unequal rates for qMN and qNM with MCMC-and a stepping stone sampler for motility traits.

**Protein**		**Multistate (*LogML*) (q01≠q10)**	**Multistate (*LogML*) *(q01=q10)***	**Log BF**	**Mean q10: loss**	**Mean q01:gain**
Filament	FliC	-1236.31922	-1279.36122	86.08400	0.90373	0.25412
	FliS	-1190.00789	-1238.22466	96.43356	1.01718	0.27361
	FliT	-801.54408	-1032.39299	461.69780	2.89199	0.06330
	FliD	-1201.70553	-1249.56069	95.71030	1.02036	0.25515
Rotor	FliG	-1501.62285	-1507.54012	11.83456	0.90254	0.57065
	FliM	-1282.70992	-1311.17141	56.92298	0.86913	0.31444
	FliN	-1326.05680	-1365.91744	79.72128	0.97301	0.30650
Stator	MotA	-710.72027	-715.04653	8.65252	0.16202	0.45206
	MotB	-1377.81916	-1376.49928	-2.63976	0.60413	0.60413
	MotC	-133.70585	-217.34157	167.27140	2.38561	0.00483
	MotD	-689.31934	-780.79494	182.95120	5.21982	0.27191
	FliA	-716.22016	-733.38602	34.33174	0.18116	0.73628
	FlhC	-658.33364	-754.56490	192.46250	2.85142	0.15293
	FlhD	-397.78680	-503.70056	211.82760	2.16876	0.06188
Type III secretion	FliH	-1583.38907	-1589.41584	12.05353	0.78106	1.28892
	FliI	-103.51505	-104.33658	1.64305	0.02074	0.02074
	FliJ	-1279.14958	-1360.92219	163.54520	1.17715	0.24889

	FliO	-1293.75425	-1371.39046	155.27240	1.13792	0.19901
	FliP	-1176.40907	-1212.72037	72.62260	0.80795	0.23681
	FliQ	-1195.92129	-1232.73389	73.62520	0.83035	0.24063
	FliR	-1185.52114	-1224.97072	78.89914	0.83879	0.23415
	FlhA	-1173.68779	-1208.28648	69.19738	0.80626	0.24361
	FlhB	-1183.56344	-1217.71889	68.31088	0.81330	0.24324
	FlhE	-241.15431	-337.93076	193.55290	6.16114	0.04887
MSP-and L rings	FliF	-1181.44873	-1217.41895	71.94044	0.80826	0.23886
	FlgI	-913.02227	-1027.67297	229.30140	0.93715	0.05979
	FlgA	-1423.41473	-1453.98837	61.14728	0.94353	0.33304
	FlgH	-842.18787	-972.78843	261.20120	0.92798	0.04762
	FliL	-1232.83343	-1270.54440	75.42194	0.89004	0.26773
H and T rings	FlgT	-1645.17804	-1716.37441	142.39275	1.67847	0.49569
	FlgO	-1136.09156	-1181.56333	90.94354	3.12123	0.41400
	FlgP	-669.65105	-707.76419	76.22628	1.49630	0.13231
	FlgQ	-60.89570	-81.73084	41.67027	1.29738	0.00307
	MotX	-1480.69147	-1505.55385	49.72477	0.51793	1.33592
	MotY	-916.49661	-916.39461	-0.20399	0.28872	0.28872
Rod and hook	FliE	-1204.81319	-1255.13299	100.63960	0.91200	0.21355
	FliK	-1487.93757	-1557.32973	138.78430	1.28489	0.35445
	FlgB	-1808.17981	-1849.13994	81.92026	1.54182	0.58505
	FlgC	-1261.29225	-1318.24910	113.91371	0.98673	0.22182
	FlgD	-1776.49188	-1772.77320	-7.43737	0.95007	0.95007
	FlgF	-1328.83804	-1474.90444	292.13280	1.37494	0.12637
	FlgG	-1409.14140	-1538.21955	258.15631	1.56650	0.21522
	FlgJ	-1288.13235	-1313.68305	51.10140	0.83527	0.28980
	FlgE	-1306.79517	-1367.16020	120.73005	1.02110	0.22166
	FlgK	-1233.75053	-1274.29683	81.09260	0.89237	0.26066
	FlgL	-1478.32921	-1551.66187	146.66532	1.34815	0.29723
	FlgN	-1236.89244	-1308.90719	144.02949	1.42868	0.28302
Regulator	FlrA	-51.46165	-57.63526	12.34721	0.05490	27.07270

	FlgM	-879.56745	-1034.52229	309.90969	1.30375	0.07797
	FlrC	-116.83022	-123.78185	13.90326	0.02247	1.13897
	FliZ	-77.58300	-141.17030	127.17459	3.55618	0.00827
Other	FliY	-574.26291	-629.30654	110.08726	0.10687	1.21131
	RpoD	-70.13664	-77.10501	13.93674	0.08113	28.79060
	RpoN	-916.83470	-912.49329	-8.68281	0.33818	0.33818

Supplementary Table S5: Log Marginal Likelihood (log ML) estimates using the BayesTraits Multistate algorithms for equal and unequal rates for q01(gain) and q10(loss) with MCMC and a stepping stone sampler for individual flagellar proteins.

**Substructure**	**Protein**	**Comparison**	**Dependent model (*Log ML*)**	**Independent Model (*Log ML*)**	**LogBF**
Filament	FliC	Motility	-1262.71280	-2259.65168	1993.87776
	FliS	FliC	-1609.82523	-2428.14162	1636.63277
	FliT	FliC	-1887.20352	-2037.23893	300.07082
	FliD	FliC	-1576.97481	-2439.81757	1725.68551
Rotor	FliG	FliC	-1921.98074	-2737.89146	1631.82145
	FliM	FliC	-1623.90728	-2516.09482	1784.37507
	FliN	FliC	-1745.42553	-2564.63547	1638.41988
Stator	MotA	FliC	-1619.49209	-1947.20304	655.42189
	MotB	FliC	-2171.08961	-2612.84953	883.51984
	MotC	FliC	-1341.32405	-1369.54082	56.43355
	MotD	FliC	-1822.04517	-1915.64730	187.20426
	FliA	FliC	-1913.32034	-1950.04135	73.44201
	FlhC	FliC	-1802.40590	-1893.15795	181.50409
	FlhD	FliC	-1519.01125	-1633.49469	228.96686
Type 3 secretion	FliH	FliC	-2591.42687	-2818.24909	453.64443
	FliI	FliC	-1330.86220	-1339.66738	17.61036
	FliJ	FliC	-2068.58405	-2509.92630	882.68449
	FliO	FliC	-1993.05548	-2527.58780	1069.06463
	FliP	FliC	-1469.81902	-2414.03878	1888.43952

	FliQ	FliC	-1505.21939	-2429.57468	1848.71058
	FliR	FliC	-1627.99927	-2427.91456	1599.83058
	FlhA	FliC	-1488.43939	-2415.79924	1854.71971
	FlhB	FliC	-1502.95525	-2426.35114	1846.79178
	FlhE	FliC	-1453.74767	-1477.16652	46.83769
MSPand L rings	FliF	FliC	-1490.91907	-2418.64436	1855.45059
	FlgI	FliC	-1579.05268	-2149.15179	1140.19822
	FlgA	FliC	-2375.90828	-2658.61362	565.41068
	FlgH	FliC	-1475.30072	-2083.21063	1215.81983
	FliL	FliC	-1692.94741	-2474.08792	1562.28102
H and T rings	FlgT	FliC	-2857.43653	-2884.33167	53.79028
	FlgO	FliC	-2328.72413	-2370.49430	83.54033
	FlgP	FliC	-1866.36753	-1908.03772	83.34038
	FlgQ	FliC	-1293.52206	-1297.17991	7.31569
	MotX	FliC	-2662.67957	-2719.30568	113.25221
	MotY	FliC	-2133.37752	-2151.97865	37.20226
Rod and hook	FliE	FliC	-1451.36643	-2439.29563	1975.85840
	FliK	FliC	-2082.22614	-2712.73815	1261.02402
	FlgB	FliC	-2207.25112	-3044.12297	1673.74370
	FlgC	FliC	-1549.05934	-2504.72874	1911.33881
	FlgD	FliC	-2254.97238	-3013.15973	1516.37469
	FlgF	FliC	-1984.34351	-2566.27817	1163.86934
	FlgG	FliC	-2121.55607	-2647.15897	1051.20581
	FlgJ	FliC	-2256.67354	-2524.04003	534.73300
	FlgE	FliC	-1597.33315	-2539.73904	1884.81178
	FlgK	FliC	-1455.89385	-2470.06691	2028.34611
	FlgL	FliC	-1883.79719	-2710.82182	1654.04926
	FlgN	FliC	-1889.40581	-2479.26385	1179.71608
Regulator	FlrA	FliC	-1281.63765	-1287.77814	12.28098
	FlgM	FliC	-1725.17754	-2117.50901	784.66293
	FlrC	FliC	-1347.58112	-1353.45889	11.75555

	FliZ	FliC	-1292.64679	-1313.16429	41.03501
Other	FliY	FliC	-1778.49557	-1809.60357	62.21599
	RpoD	FliC	-1298.88130	-1305.03087	12.29915
	RpoN	FliC	-2085.73875	-2151.56623	131.65496

Supplementary Table S6: Log Marginal Likelihood (log ML) estimates using the BayesTraits Multistate algorithms for dependent and independent models for correlated evolution of other proteins vs FliC.

Supplementary Table S7: The list of species with mismatched motility classification as compared with the phenotypic data curated from published literature [13, 21].

Supplementary Phylogeny S1: Phylogenetic tree of 4125 species pruned from reference GTDB tree in Nexus format.

Supplementary Phylogeny S2: Phylogenetic tree of 100 representative genera pruned from reference GTDB tree using one species per genus in Newick format.
